# Identification and Feeding Characterization of *Sterkiella histriomuscorum* (Protozoa, Ciliophora, Hypotrichia) Isolated from Outdoor Mass Culture of *Scenedesmus dimorphus*

**DOI:** 10.3390/microorganisms13051016

**Published:** 2025-04-28

**Authors:** Mengyun Wang, Pei Chen, Hongxia Wang, Qiong Deng, Xiaonan Zhang, Guoqing Yuan, Mixue Jiang, Lingling Zheng, Zixuan Hu, Zemao Gu, Denis V. Tikhonenkov, Yingchun Gong

**Affiliations:** 1 Institute of Hydrobiology, Chinese Academy of Sciences, Wuhan 430072, China; 13207148099@163.com (M.W.); hongxiawang@ihb.ac.cn (H.W.);; 2 State Key Laboratory of Freshwater Ecology and Biotechnology, Institute of Hydrobiology, Chinese Academy of Sciences, Wuhan 430072, China; 3 Hubei Key Laboratory of Fish Resources Protection of Three Gorges Project, Chinese Sturgeon Research Institute of CTG, Yichang 443100, China; 4 College of Life Sciences and Technology, Huazhong Agricultural University, Wuhan 430070, China; 5 Freshwater Algae Culture Collection at the Institute of Hydrobiology, Wuhan 430072, China; 6 Papanin Institute for Biology of Inland Waters, Russian Academy of Sciences, 152742 Borok, Russia

**Keywords:** *Sterkiella histriomuscorum*, algivory, feeding, excystment, SSU rRNA

## Abstract

Herbivorous protistan grazers are ubiquitous and abundant in marine and temperate freshwater environments. However, little is known about the algivorous ciliates and their feeding habits in outdoor mass algal cultures. In this study, we report on one hypotrich ciliate, identified as *Sterkiella histriomuscorum*, from the outdoor mass culture of *Scenedesmus* in Arizona, USA. A long-term field survey revealed that this species often occurs in *Scenedesmus* culture in spring and summer, and can graze very heavily on *Scenedesmus* cells. By isolating *Sterkiella* cells and then observing them via light microscopy and electron microscopy, detailed information about the morphology, ultrastructure, excystment process, and feeding characteristics of the ciliate was obtained. Specifically, it seems that *S. histriomuscorum* has a range of different strategies for excystment, and the sharp change in the ion concentration in the environment around the cyst results in osmotic shock, which likely facilitates the excystment. Feeding experiments revealed that *S. histriomuscorum* preferred to graze on chlorophytes as well as the diatom *Phaeodactylum tricornutum* and had no interaction with chrysophytes or cyanobacteria. Molecular phylogenetic analysis based on the SSU rRNA gene sequence indicated that both the genus *Sterkiella* and the species *S. histriomuscorum* are non-monophyletic. The information obtained from this study will help advance our understanding of the biodiversity and ecological function of *S. histriomuscorum*, and will also be very useful in the development of early warning systems and control measures for preventing or treating this contaminant in microalgal mass cultures.

## 1. Introduction

Herbivorous protistan grazers are ubiquitous and abundant in marine and temperate freshwater environments, and they are diverse not only in terms of taxonomy but also in terms of size and feeding behavior [1]. Grazing is recognized as a process structuring phytoplankton communities that plays a key role in carbon cycling and regeneration [2]. In recent years, increasing attention has been given to the utilization of algal biomass production, such as in wastewater bioremediation, carbon capture, and the production of animal feed and biofuels from algal biomass [3]. However, the use of algal biomass has been hampered by difficulties in cultivating the alga on a commercial scale, caused mainly by contamination and grazing by protist predators [4]. Most herbivorous ciliates belong to the classes Spirotrichea, Nassophorea, and Oligohymenophorea [1], which are highly diverse [5] and play important roles in shaping planktonic community structures in pelagic freshwater ecosystems [6]. However, the diversity and distribution of herbivorous ciliates in mass algal culture systems are unknown or not clear.

Hypotrich ciliates (Spirotrichea) are considered one of the most confusing and divergent groups of protists [7], and most of them are observed to have herbivorous habits [8,9]. *Sterkiella histriomuscorum* is a cosmopolitan herbivorous hypotrich ciliate [10]. Many studies have been carried out on the taxonomy [11,12,13,14,15,16] and encystment–excystment cycle [17,18] of *S. histriomuscorum*; however, many aspects of its ecology and biology remain unclear. In terms of taxonomy, Kahl provided the original description for the species as early as 1932, and thirteen species of *Sterkiella* have been reported so far [16]. However, the evolutionary relationships among different strains of *S. histriomuscorum* or different species of *Sterkiella* are still complex and confusing [16], as they were proven to be non-monophyletic on the basis of molecular phylogenetic analysis [11,12,13,14,15,16], despite their well-outlined morphological characteristics and highly stable ontogenetic processes [19].

To elucidate the identification characteristics of herbivorous protists, it is also essential to understand their feeding characteristics. Most herbivorous protists are known to be very selective consumers that can recognize various traits of their potential prey organisms [20], and it is generally assumed that selection by protists occurs during capture and ingestion [21]. Recently, preferential grazing and repackaging of small polyethylene microplastic particles by the ciliate *Sterkiella* sp. have been reported [22]. As most protists select food based on particle size, not by “choice” [23], it is not a good nutritional choice that *Sterkiella* sp. preferred microplastics when they are the right size; the feeding selection of *Sterkiella* probably takes place in the process of digestion. Moreover, few studies have focused on the grazing of *Sterkiella* on phytoplankton, which play an important functional role in ecosystems [2].

Therefore, in the present study, we carried out an in-depth investigation of a new strain of *Sterkiella histriomuscorum*, isolated from an outdoor mass culture of *Scenedesmus dimorphus*. Here, we present a detailed description of its morphology, excystment process, feeding characteristics, and phylogenetic position on the basis of SSU rRNA gene sequence data.

## 2. Materials and Methods

### 2.1. Isolation and Cultivation

The algal cultures of *Scenedesmus dimorphus* (UTEX 1237) were grown in outdoor raceway ponds (ca. 600 L, 0.20 m depth) with modified BG-11 culture medium [24,25] on the Mesa campus of Arizona State University (33°18′15″ N, 111°40′23″ W) in the southwestern United States for three years (March to October; 2010–2012). The culture in the pond was circulated at an average surface velocity of 0.2–0.3 m s^−1^ using a steel paddlewheel with 6 blades and was aerated using compressed air with 1.5% CO_2_ to adjust and maintain the pH within the range of 6.5 to 7.5 during the whole cultivation. The temperatures in the raceway ponds were not regulated and were affected by the local weather. One batch of cultivation lasted 5–7 days. The climate is primarily dry and hot, with very low rainfall throughout the year, and is extremely hot in the summers. According to the Arizona Meteorological Network (http://ag.arizona.edu/azmet/22.htm, accessed on 1 January 2013), the air temperature ranged from 8.3 to 36.1 °C (47–97 °F). A survey of protozoan contamination was conducted every day during cultivation. The ciliate *S. histriomuscorum*, which was first found in algal cultures of *S. dimorphus* in 2011, was isolated with a glass micropipette under a dissecting microscope and cultured in Petri dishes at 25 °C with *Chlorogonium elongatum* as a food source [26].

### 2.2. Feeding Impact Analysis

To evaluate the ability of the ciliate to graze on *Scenedesmus* cells, the ciliate cells were picked from the ciliate culture by gentle pipetting under a dissecting microscope and maintained in 5 mL of algae-free BG11 medium at room temperature. In order to simulate the practical conditions of outdoor algal culture [27,28], the initial concentration of *Scenedesmus* cells was 1.2 × 10^6^ cells/mL, while the ciliate density of *Sterkiella* was set at 120 ind./mL, and the ratio was 10,000:1. After 3 h of starvation, approximately 600 ciliate cells were introduced into each well of a 6-well microplate, each well containing 5 mL of *Scenedesmus* culture, and incubated in the dark at 21 °C for 24 h with continuous gentle shaking at 120 rpm. A 1 mL sample of the ciliate-algal mixture was taken at 0 h and 24 h and preserved with Lugol’s solution (1%, final concentration) for enumeration of both *Sterkiella* and *Scenedesmus* cells. In our preliminary experiment, Lugol’s solution was not observed to lyse *Sterkiella* cells. The clearance rate (Cr) was used to evaluate the grazing impact on the growth of *Scenedesmus* using the following formula: $Cr=\frac{A1-A2}{C}$, where “A1” and “A2” are the cell concentrations of the alga *Scenedesmus* at 0 h and 24 h, respectively, and “C” is the cell concentration of the ciliate *Sterkiella* at the end of the experiment. The cells were counted using a hemocytometer, with each sample being counted three times to determine the mean cell concentration.

Moreover, the time setting of the experiment should be clarified. Based on our observation, our strain will soon form cysts when there is no food available. If more than 3 h, most of the ciliates will form cysts, so in our study, we only took 3 h to starve the ciliates. Similarly, in our preliminary experiment, the grazing rate of the ciliate was very high. When supplied with suitable food, the ciliate could clear almost all the algal cells in one day. Therefore, in our study, we only conducted short-term experiment. The aim of our study was to measure the grazing ability, and one day is enough for evaluation.

### 2.3. Light Microscopy

Light micrographs of live cells and cysts were taken with a bright-field and differential interference contrast microscopy, using a Zeiss Axioplan microscope (Carl Zeiss, Hallberg-moos, Germany). Excystment was induced by incubating the *Sterkiella* cysts, which were formed from vegetative cells due to the lack of sufficient algae as food in the cultures. Then, about twenty cysts were picked up and immersed in distilled water at room temperature for more than 10 min. To reveal the infraciliature, the *Sterkiella* cells were stained with protargol [29]. Counts and measurements of the morphological characteristics of the protargol-stained specimens were performed at a magnification of ×1000.

### 2.4. Scanning and Transmission Electron Microscopy

For scanning electron microscopy (SEM), hundreds of *Sterkiella* cells or cysts were picked out with a micropipette under a dissecting microscope and collected in 1.5 mL centrifuge tubes. The cell suspension was fixed with an equal volume of a mixture of osmium acid (2%) and saturated mercuric chloride (volume ratio 1:1). Using the method by Gong et al. [30], one drop of pellet from the bottom of the tube was placed onto a glass coverslip coated with 0.1% poly-L-lysine and left to stand for 30 min at 4 °C. The adherent cells were washed three times with phosphate-buffered saline (PBS) and subsequently fixed with 1% osmium tetroxide (OsO_4_) in PBS for 1 h at room temperature. After three rinses in ultrapure water, the samples were dehydrated in a graded acetone series using isoamyl acetate and then dried with a critical-point dryer HCP-2 (Hitachi, Tokyo, Japan) using liquid carbon dioxide. Coverslips with attached cells were mounted on an aluminum stub and coated with approximately 15 nm of gold–palladium in an E102 ion sputter (Hitachi, Japan). The specimens were examined with a Su8010 scanning electron microscope (Hitachi, Japan) operated at 15 kV.

For transmission electron microscopy (TEM), the cells were harvested by gentle centrifugation (3000× *g* for 10 min; Eppendorf, MiniSpin, Hamburg, Germany) and fixed with PBS buffer (ca. pH 7.2–7.4) containing 2.5% glutaraldehyde overnight at 4 °C. After washing in PBS, the cell samples were post-fixed with 1% OsO_4_ in PBS for 2 h at 4 °C. Following stepwise acetone dehydration and infiltration with Spurr’s epoxy resin, the cell samples were embedded and polymerized in Spurr’s epoxy resin at 60 °C for 48 h. Ultrathin sections (70 nm) were cut using a Leica EM UC-7 microtome and double-stained with 2% uranyl acetate and Sato’s lead citrate [31]. The specimens were examined with a Hitachi 7700 transmission electron microscope, operated at 80 kV.

### 2.5. Ability of the Ciliate to Feed on Other Microalgae

To explore the grazing ability of *Sterkiella histriomuscorum*, 17 different strains of algae were selected as prey, including 10 chlorophytes, 2 chrysophytes, 1 diatom, and 4 cyanobacteria (Table 1). All species were obtained from the Freshwater Algae Culture Collection (FACHB) and the Algae Biotechnology and Center for Microalgal Biotechnology and Biofuels (CMBB) at the Institute of Hydrobiology, Chinese Academy of Sciences, Wuhan, China.

The experiment was carried out in a 6-well plate with a working liquid volume of 6 mL for one week at 25 °C, and the plates were covered with foil in a dark environment. For the control group, only algae were added. For each algal strain, tests were conducted in triplicate. The initial concentration of algal cells in the treatment group and control group was set as (6.0 ± 0.5) × 10^6^ cells/mL, and the initial concentration of the ciliate in the treatment group was set as (2.0 ± 0.1) × 10^2^ cells/mL. A 1 mL sample of the ciliate-algal mixture was taken daily and preserved with Lugol’s solution (1%, final concentration) for enumeration of both *Sterkiella* and algal cells. The cells were counted using a hemocytometer for algae and a 0.1 mL counting chamber for ciliates, with each sample being counted three times to determine the mean cell concentration.

In addition, cultures were sampled daily for observation and microphotography under a light microscope (BX53, Olympus, Tokyo, Japan) to determine the following: (i) whether the ciliates were feeding, (ii) whether ciliate populations were growing (assessed qualitatively), and (iii) whether the growth of the ciliates was persistent (i.e., the ciliates did not die before the food source was exhausted).

### 2.6. DNA Extraction, PCR, Sequencing, and Phylogenetic Analysis

Approximately one hundred *Sterkiella* cells were collected, and the genomic DNA was extracted with the RED_Extract-N-Amp^TM^ Tissue PCR Kit (Sigma, Saint Louis, MO, USA) following the manufacturer’s instructions. The small subunit (SSU) rRNA gene was amplified by polymerase chain reaction (PCR) with the universal primers EUK-SSUA (5′-AACCTGGTTGATCCTGCCAGT-3′) and EUK-SSUB (5′-TGATCCTTCTGCAGGTTCACCTAC-3′) [32]. The amplification parameters were as follows: initial denaturation at 94 °C for 10 min, then five cycles of denaturation for 1 min at 94 °C, primer annealing for 1.5 min at 50 °C, and extension for 2.5 min at 72 °C, followed by 30 cycles in the same manner, but with the annealing temperature increased to 56 °C, and a final extension step at 72 °C for 10 min. The PCR product was purified using the High-Pure PCR Product Purification Kit (Omega, Bio-Tek, Norcross, GA, USA), inserted into the pMD-19T Simple Vector (TaKaRa Biotechnology Ltd., Dalian, China), and transformed into competent *Escherichia coli* DH5α. Five recombinant plasmids were sequenced in both directions using an ABI PRISM^®^ 3730 DNA sequencer (Applied Biosystems Inc., Foster City, CA, USA). The sequences were assembled using the SeqMan software of DNAStar (DNASTAR Inc., Madison, WI, USA).

The SSU rRNA gene sequence of the *S. histriomuscorum* strain ASU-2012 was deposited in GenBank under the accession number KX355209. To assess the phylogenetic position of the ciliate we isolated, 42 SSU rRNA gene sequences from the Hypotrichia and Oligotrichia groups were retrieved from the GenBank database, in addition to our new sequence, aligned using the Clustal X version 1.8 software [33], and refined by GBLOCKS [34]. The final alignment that was used for subsequent phylogenetic analyses comprised 1755 positions. The optimal evolutionary model of sequence evolution was selected using ModelTest3.7 with the Akaike information criterion [35] as the general time reversible model (GTR + I + G). Maximum likelihood (ML) analysis was performed with the online software PhyML 3.0 (http://www.atgc-montpellier.fr/phyml/, accessed on 1 December 2024) [36]. Bootstrap support values were obtained after 1000 replicates. Bayesian inference (BI) analysis was conducted with MrBayes version 3.1.2 [37] using the same evolutionary model. Markov chains were run for 1,000,000 generations and sampled every 100 generations, and the first 2500 trees were discarded as burn-in. A consensus tree was generated with the remaining trees to determine the posterior probabilities at the different nodes. The trees were edited and annotated in MEGA 5 [38], and two Oligotrichia species were used as outgroup.

The statistical probability of the hypothesis that *Sterkiella* is a monophyletic lineage was evaluated using AU tests [39], as described by Zhang et al. [40].

### 2.7. Terminology

For general and specific terms, see Lynn [41] and Berger [42]. Details of the ciliate oral apparatus are according to Foissner and Al-Rasheid [43].

## 3. Results

### 3.1. Feeding Impact of the Ciliate on the Growth of Scenedesmus

**Figure 1:**
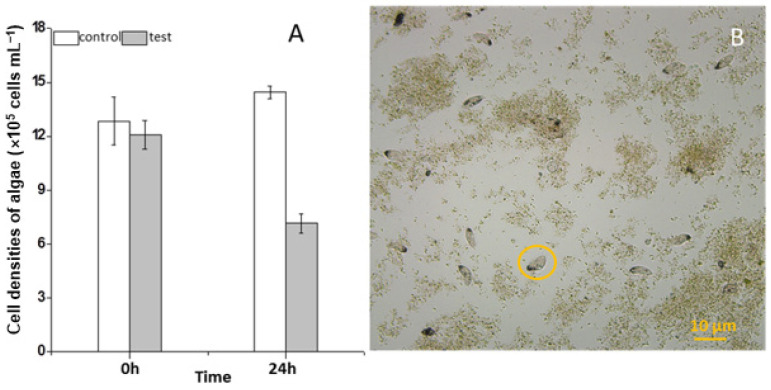
Impact of feeding by *Sterkiella histriomuscorum* on the growth of *Scenedesmus*. (**A**) Changes in the cell concentration of *Scenedesmus* after grazing by *S. histriomuscorum* for 24 h in the dark. (**B**) The destroyed Scenedesmus culture, contaminated by a large number of *S. histriomuscorum*, with many Scenedesmus cells aggregated with debris into large lumps. The orange circle indicates the ciliate *S. histriomuscorum*.

During the three years (2010–2012) of the survey, the ciliate *S. histriomuscorum* strain ASU-2012 was often found to contaminate the outdoor mass cultures of *Scenedesmus* in spring and summer. The trophozoites usually occur on the third or fourth day after the *Scenedesmus* cells are inoculated into the culture and exhibit a strong ability to graze on algae once they have grown. Our laboratory experiment using *S. histriomuscorum* isolated from these cultures revealed that after co-culture with *Scenedesmus* for 24 h, the concentration of the unicellular alga *Scenedesmus* was halved and reduced to 5.0 × 10^5^ cells/mL (Figure 1A), and the concentration of the ciliate was doubled to 240 cells/mL, indicating that one *S. histriomuscorum* cell could capture approximately 2.0 × 10^3^ unicellular *Scenedesmus* cells in one day, and that the clearance rate of *Sterkiella* on *Scenedesmus* was about 87 cells per hour. Microscopy revealed that *Sterkiella* grazed *Scenedesmus* cells very efficiently (Movie S1), and many *Scenedesmus* cells were captured inside the cells of *S. histriomuscorum*, whereas many other *Scenedesmus* cells aggregated with debris into large clumps (Figure 1B), resulting in a decrease in the algal growth rate and deterioration of the algal cultures. When no unicellular or dispersed algal cells were available for food, the trophozoites soon transformed into cysts. Compared with those of the ciliates in the outdoor algal cultures, the behavior of the ciliates in the lab cultures did not differ much; both of them moved very quickly and grazed very fast. A minor difference was that the ciliates in the outdoor cultures were more active.

### 3.2. Morphological Observation

#### 3.2.1. Light Microscopy of *Sterkiella histriomuscorum*

**Figure 2:**
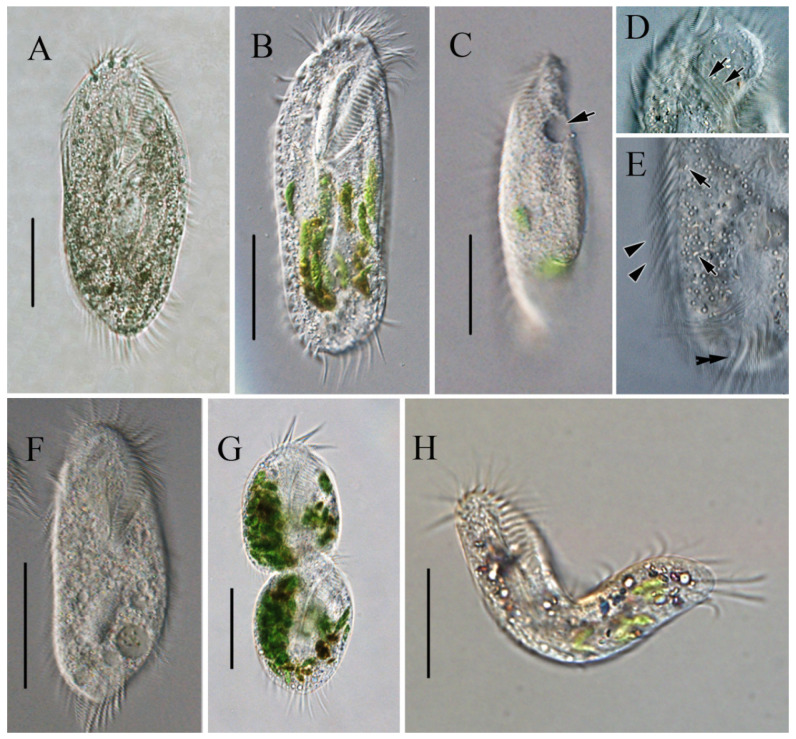
Morphology of *Sterkiella histriomuscorum* in vivo under light microscopy. (**A**,**B**) Ventral view of representative specimens. (**C**) Side view—arrow shows one contractile vacuole located in the anterior 2/5 of the body. (**D**) Undulating membrane, as shown by arrows; (**E**) Endoplasm, arrows showing some cytoplasmic granules, arrowheads indicating left marginal cirrus, and double-headed arrow indicating transverse cirri. (**F**) Small-sized colorless individual. (**G**) Individual in transversal division; (**H**), Flexible individual. Scale bars: 50 µm.

In vivo, the cells are approximately 90–150 × 45–65 μm, slightly flexible and elliptical, generally with the anterior end slightly pointed and the posterior end bluntly rounded (Figure 2A,B). One contractile vacuole was located near the left margin in the anterior 2/5 of the body, partly overlapping with the adoral zone of membranelles (AZM; Figure 2C), contracting at intervals of about 20 s. The undulating membrane, the left marginal cirrus, and noticeable transverse cirri were observed (Figure 2D,E). Cortical granules were absent. The cytoplasm was colorless, with many cytoplasmic granules (Figure 2E, ca. 2–4 μm across). Unlike most cells, some cells do not form cysts when food supplies are insufficient for cell growth and division but survive and decrease in size, with smaller cells being morphologically similar to normal cells but with a colorless to grayish endoplasm (Figure 2F). The cells divided transversely (Figure 2G). The cells are usually rigid, but small cells that appear slightly flexible are occasionally observed (Figure 2H). Locomotion occurs by fast crawling on a substrate, occasionally swimming forward with rotation around the main body axis.

**Figure 3:**
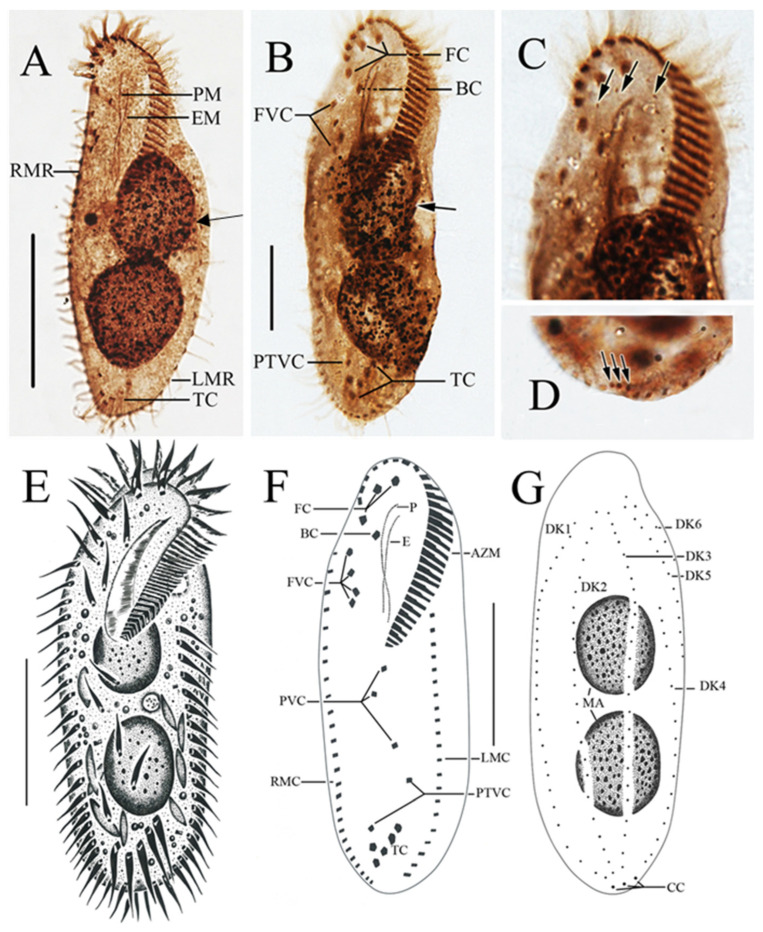
Morphology of *Sterkiella histriomuscorum* after protargol impregnation (**A**–**D**) and by drawing (**E**–**G**). (**A**,**B**) Ventral view, arrows pointing to the macronucleus in division. (**C**) Detailed view of the anterior part of the body, arrows indicating dorsal kineties. (**D**) Detailed view of the posterior part of the body, arrow indicating caudal cirri. (**E**) Ventral view of a typical individual. (**F**,**G**) Ventral and dorsal view of the infraciliature of the same specimen. BC: Buccal cirri; EM: Endoral membrane; FC: Frontal cirri; FVC: Frontoventral cirri; LMR: Left marginal row; PM: Paroral membrane; PTVC: Pretransverse ventral cirri; RMR: Right marginal row; TC: Transverse cirri. Scale bars: 50 µm.

The ciliates were also observed after staining with protargol (Figure 3; Table 2). The adoral zone is composed of 28–38 membranelles (Figure 3A,B,E,F) with cilia about 15–18 µm long and the bases of the membranelles about 15 µm long. Undulating membranes are in the typical *Oxytricha* pattern: the endoral membrane is shorter than the paroral membrane, and both membranes intersect in the middle, with a single buccal cirrus located nearby on the anterior end close to the anterior 1/3 of the undulating membranes (Figure 3A,B,E,F). Three slightly enlarged frontal cirri with cilia about 10–12 µm long, and four frontoventral cirri closely arranged in an irregular V-shaped row were located to the right of the buccal field (Figure 3B,F). The ventral cirri are arranged in a 2:1:2 pattern: three postoral ventral cirri with the rear one positioned distant from the anterior two, whereas the two pretransverse cirri are located near the transverse cirri (Figure 3F). Five relatively strong transverse cirri in the J-shaped row were located near the posterior end of the venter (Figure 2E and Figure 3B,F), with cilia approximately 16–18 µm long. The left and right marginal rows are composed of 18–25 and 22–27 cirri, respectively, and are slightly separated at the posterior end of the cell, with cilia approximately 12–16 µm long (Figure 3A,E).

The dorsal ciliature is composed of six dorsal kineties, comprising four dorsal kineties and two dorsomarginal kineties (Figure 3C,G): dorsal kineties 1, 2, and 3 almost reach the length of the cell; kinety 4 is slightly shortened anteriorly, starts at the anterior 1/3 of the cell length, and terminates at the posterior end of the cell; the rightmost two dorsomarginal kineties 5 and 6 commence near the anterior end of the cell, dorsal kinety 5 terminates at the equatorial level and is usually composed of 13 dikinetids, whereas dorsal kinety 6 terminates at 1/4 of the cell length and is usually composed of six dikinetids. Three conspicuous caudal cirri with cilia about 12–16 µm long, each located at the posterior end of dorsal kineties 1, 2, and 4 (Figure 3D,G). Two ellipsoidal macronuclear nodules, 24–42 µm long (Figure 3A,B,E,G).

#### 3.2.2. Electron Microscopy

**Figure 4:**
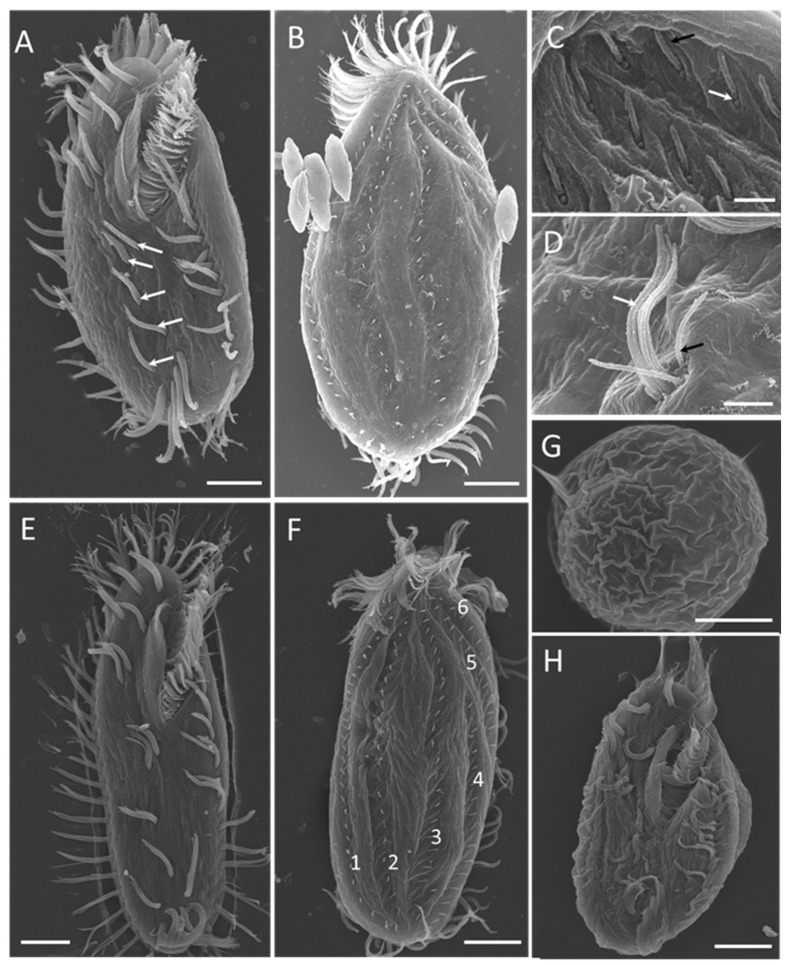
Morphology of *Sterkiella histriomuscorum* under a scanning electron microscope. (**A**) General ventral view of a mature individual, with arrows marking the ventral cirri arranged in a “2:1:2” pattern. (**B**) General dorsal view of a mature individual. (**C**) Detail of dorsal bristles, composed of two kinetosomes, one with a short cirrium (black arrow), another one is barring (white arrow). (**D**) Detail of ventral cirri, composed of two short cirri (black arrow) and about six long cirri (white arrow). (**E**) General ventral view of a young excysted individual. (**F**) General dorsal view of a young excysted individual, with 1–6 indicating the arrangement of dorsal kineties. (**G**) One-week-old resting cyst with a wrinkled surface. (**H**) Newborn individual from a cyst. Scale bars = 10 μm for (**A**,**B**,**E**–**H**); Scale bars = 2 μm for (**C**,**D**).

Scanning electron microscopy (Figure 4) revealed more detailed information about the ventral (Figure 4A,E) and dorsal (Figure 4B,F) views of individuals at different stages of the life cycle. The 2:1:2 pattern of ventral cirri was clearly shown for mature individuals (Figure 4A) and young excysted individuals (Figure 4E). The six dorsal kineties of the dorsal ciliature were obvious in the young excysted individual shown in Figure 4F. Each ventral ciliature complex was composed of 8 kinetosomes, two short cilia, and six long cilia (Figure 4D), whereas each dorsal ciliature complex was composed of two kinetosomes, only one of which had a cilium (Figure 4C).

**Figure 5:**
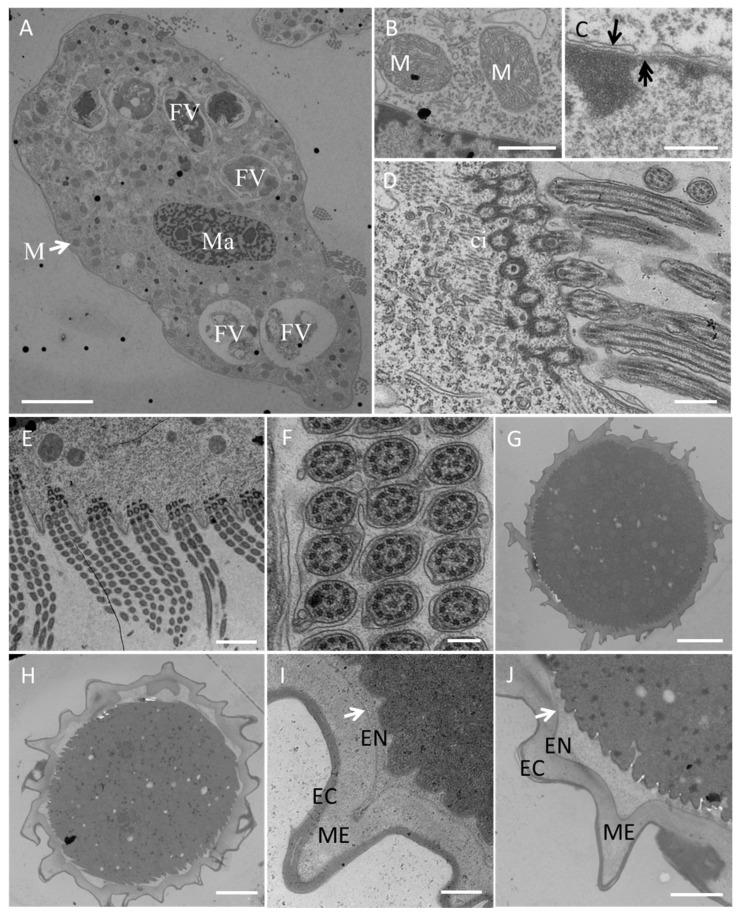
Transmission electron microscopy of a *Sterkiella histriomuscorum* cell (**A**–**F**) and cyst (**G**–**J**). (**A**) Section of a whole vegetative cell, showing the structures of the macronucleus (Ma) and mitochondrion (M), which are shown in detail in (**B**), and food vacuoles (FV); scale bar = 10 μm. (**B**) Detail of mitochondrion (M); scale bar = 200 nm. (**C**) Substructure of the macronuclear membrane, with the arrow and double arrow marking the nuclear membrane and nuclear pore, respectively; scale bar = 200 nm. (**D**) Longitudinal view of the AZM and a transverse section of a cirrus (ci); scale bar = 500 nm. (**E**), Transverse view of the AZM showing the regular arrangement of three rows of cilia; scale bar = 2 µm. (**F**) Cross section of cilia in (**E**), showing that the cilium has two central microtubule singlets and nine outer doublets; scale bar = 500 nm. (**G**) Ultrastructure of mature cyst; scale bar = 5 µm. (**H**) Ultrastructure of the cyst before excystment; scale bar = 5 µm. (**I**) Detail of the cell wall of mature cyst, arrow showing the position of the inner layer of the endocyst; scale bar = 500 nm. (**J**) Detail of the cell wall before excystment, arrow showing the position of inner layer of the endocyst; scale bar = 500 nm. EC, Ectocyst; ME, Mesocyst; EN, Endocyst.

Transmission electron microscopy (Figure 5) revealed numerous large digestive vacuoles at different stages of the digestive process and showed that one food vacuole can contain one or two algal cells (Figure 5A). Many mitochondria were distributed throughout the cell (Figure 5A,B). The macronucleus with a nuclear pore (Figure 5C) was located in the center of the cell body (Figure 5A). The prominent AZM was composed of a series of transverse linear arrangements, each comprising three rows of cilia (Figure 5D,E) with a “9 + 2” microtubule structure (Figure 5F).

### 3.3. Encystment and Excystment Processes

**Figure 6:**
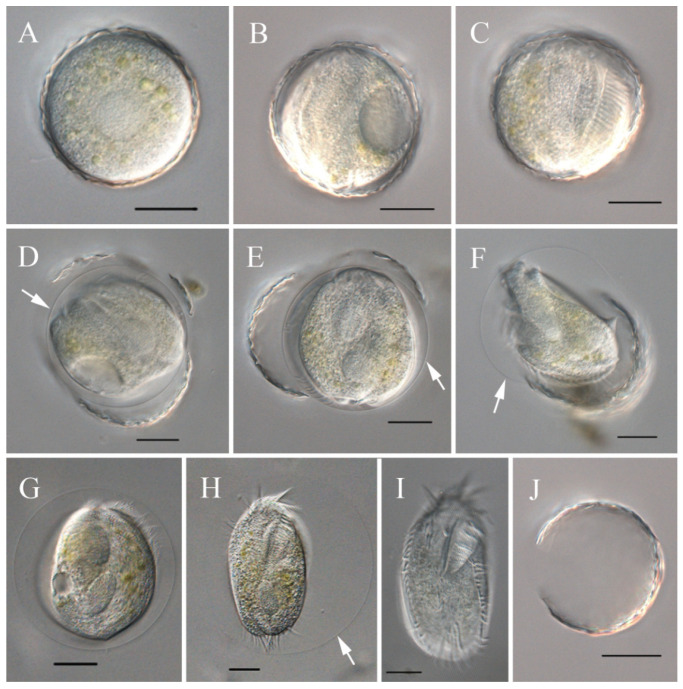
Excystment process of *Sterkiella histriomuscorum* from observation in vivo. (**A**) Cyst before excystment. (**B**,**C**) Cyst starts to excyst. (**D**) Cyst wall is breaking. (**E**,**F**) Young individual is struggling to move out of the wall. (**G**,**H**) Young individual is struggling to move out of the membrane and extending its body, with the arrow showing the cyst wall. (**I**) Young individual, just emerged due to rapid movement, is much paler than the cell in (**H**). (**J**) Empty cyst wall. All the arrows indicate the cyst wall. Scale bars = 20 μm.

The process of encystment and excystment was revealed via electron microscopy (Figure 4G,H and Figure 5G–J) and light microscopy (Figure 6).

On the basis of our observations, *S. histriomuscorum* very often forms non-sticky cysts when food supplies are lacking. The cyst has a wrinkled surface (Figure 4G) and is approximately 50 µm in diameter (Figure 5G,H). The cyst wall consists of three layers (Figure 5I,J): an outer layer of the ectocyst (EC), an inner layer of the endocyst (EN), and a mesocyst layer in the middle (ME). For normal cysts, the outer layer was much thicker than the inner layer (Figure 5I). As excystment proceeded, the outer layer became thinner, whereas the inner layer became larger and separated from the cytoplasm (Figure 5J).

Unlike encystment, excystment seldom occurs, even when plenty of food is provided. However, surprisingly, we found that excystment could be induced when the cysts were immersed in distilled water for more than 10 min. The process was visualized by light microscopy (Figure 6). Dormant cysts were covered by a compacted wall (Figure 6A), and after the cysts were incubated in distilled water for about 10 min, the cytoplasm began to flux and form a gap in the cyst wall (Figure 6B); 40 min later, the adoral zone of the membranelles appeared, and cilia beating occurred (Figure 6C); in the next 20 min, a cell body formed and moved rapidly within the cyst, surrounded by the membrane (Figure 6D,E); the cell then sheds the cyst wall and moves around with the enveloping membrane for 15–20 min (Figure 6F–H); and finally, within 5 min, the membrane disappeared, and a small ciliate specimen came out (Figure 6I), leaving the empty cyst wall (Figure 6J). Newly excysted cells were usually elliptical or obovoid in shape, with crisped cilia, slimmer than the cultured bodies (Figure 4H), and with colorless to grayish cytoplasm (Figure 6I).

### 3.4. Feeding Selectivity

The results showed that *S. histriomuscorum* preferred to graze on chlorophytes. It was found that *S. histriomuscorum* can also feed on the diatom *Phaeodactylum tricornutum* and grow rapidly (Table 1). On the basis of the ability of *S. histriomuscorum* to feed on prey, the 17 microalgal strains were divided into three groups. The first group of strains, referred to as “most suitable/rapid growth (++)”, supported rapid growth of the *S. histriomuscorum* population. This group contained seven microalgal/cyanobacterial strains, including six chlorophytes (*Chlorella vulgaris*, *C. pyrenoidosa*, *C. sorokiniana* CGMCC11801, *Scenedesmus acuminatus*, *Chlorogonium elongatum*, and *Chlamydomonas reinhardtii*) and one diatom (*P. tricornutum*). The ciliate population feeding on representatives of this algal group significantly increased in abundance in a short period of time and maintained a high population density by the end of the experiment.

The second group of algal strains, referred to as “suitable/limited growth (+)”, also served as food organisms but was less able to support the growth of the population of *S. histriomuscorum*. This group included only two strains of chlorophytes (*Chlorella sorokiniana* CMBB146 and FACHB-275). At the beginning of the experiment, the ciliates were able to feed, but as the experiment progressed, the abundance of ciliates gradually decreased, and they formed cysts.

The representatives of the third group of algae, referred to as “not suitable/no growth (-)”, were not used as food objects by the ciliates, and the ciliates showed no signs of interaction with the algae of this group. This group included two chlorophytes, two chrysophytes, and four cyanobacteria. When these algae were used as food, the ciliate population abundance significantly decreased, and by the end of the experiment, there were almost no ciliates remaining.

### 3.5. Molecular Phylogenetic Analysis

The obtained SSU rRNA gene sequence of the *S. histriomuscorum* strain ASU-2012, with a length of 1767 base pairs and a GC content of 45.10%, has been deposited in the GenBank database under the accession number KX355209. Including this sequence, six SSU rDNA sequences of *S. histriomuscorum* are available in GenBank. They differ from each other by 0–20 bp, with sequence identities ranging from 0.985 to 1.000 (Table S1). In total, there are 20 SSU rDNA sequences from ten species of the genus *Sterkiella* (*S. cavicola*, *S. histriomuscorum*, *S. multicirrata*, *S. nova*, *S. paratricirrata*, *S. sinica*, *S. tetracirrata*, *S. tricirrata*, *S. zhangi*, and *S. subtropica*) in GenBank. They differ from each other by 0–36 bp, with sequence identities ranging from 0.976 to 1.000 (Table S1).

**Figure 7:**
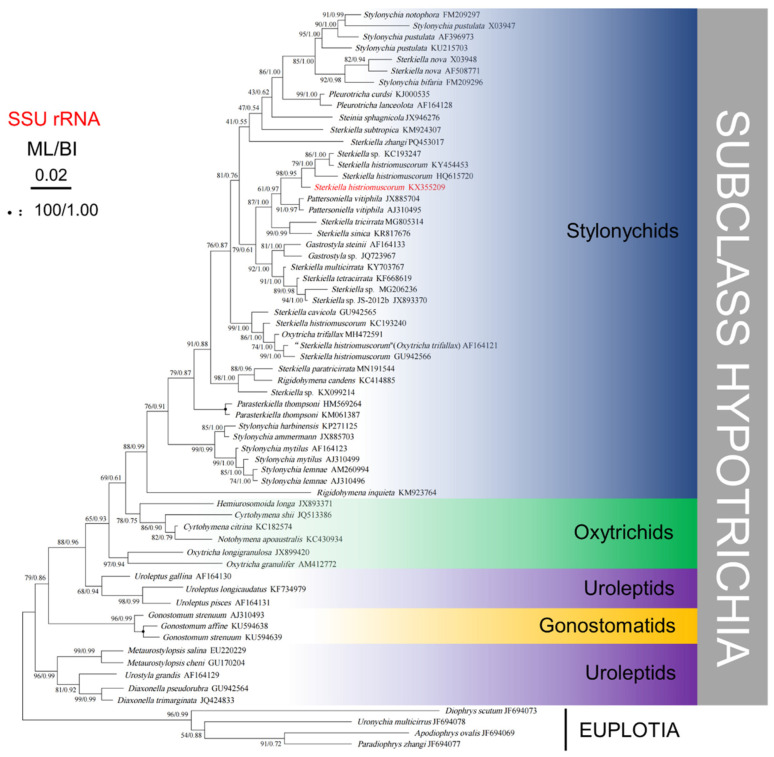
Phylogenetic tree generated from maximum likelihood and Bayesian analysis of SSU rRNA gene sequences of *Sterkiella histriomuscorum* and related ciliates. The Genbank accession numbers are listed adjacent to the species names. Bootstrap support values after 1000 replicates and Bayesian posterior probabilities are indicated at nodes when they are above 50% and 0.70, respectively. The black circles represent support values at or above 90%/0.95.

Phylogenetic trees based on SSU rRNA gene sequences and generated using BI and ML analyses were found to have similar topologies; therefore, only the ML tree is presented (Figure 7). Phylogenetic analyses consistently placed the new population of *S. histriomuscorum* (strain ASU-2012) within the clade of Stylonychids, which clustered strongly with another two populations of *S. histriomuscorum* (HQ615720 and KY454453) and one unidentified strain of *Sterkiella* sp. (KC193247) (98% ML, 0.95 BI). Another three populations of *S. histriomuscorum* located in different clades and grouped with *Sterkiella cavicola* (99% ML, 1.00 BI) and *Oxytricha trifallax* (86% ML, 1.00 BI). All ten *Sterkiella* species (twenty sequences) did not cluster together but were distributed into four main clades, sometimes containing species from other genera. More specifically, (1) two populations of *Sterkiella nova* as well as *S. subtropica* and *S. zhangi* located in the top clade within stylonychids, and some species from other genera, including *Stylonychia*, *Pleurotricha*, and *Steinia*, also grouped in one clade; (2) three populations of *S. histriomuscorum*, *S. tricirrata*, *S. sinica*, *S. multicirrata*, *S. tetracirrata*, and three undefined congeners *Sterkiella* sp. (KC193247), *Sterkiella* sp. (MG206236), and *Sterkiella* sp. JS-2012b (JX893370) formed a large clade, and some species from other genera, including *Pattersoniella* and *Gastrostyla*, grouped in a clade; (3) three populations of *S. histriomuscorum*, *S. cavicola*, and one species from another genus (*O. trifallax*) located in a clade (99% ML, 1.00 BI); and (4) *S. paratricirrata*, one undefined *Sterkiella* sp. (KX099214), and one species from another genus (*Rigidohymena candens*) formed the last clade (98% ML, 1.00 BI). The AU tests rejected the possibility that the genus *Sterkiella* or the species *S. histriomuscorum* has a monophyletic lineage.

## 4. Discussion

### 4.1. Identification of S. histriomuscorum

According to Berger (1999) [7], the genus *Sterkiella* contains eight nominal species: *S. cavicola* [44], *S. admirabilis* [45], *S. histriomuscorum* [44], *S. nova* [46], *S. terricola* [7], *S. thompsoni* [47], *S. tricirrata* [7], and *S. quadrinucleatus* [7]. Among them, *S. histriomuscorum* has been considered a sibling species complex with *S. nova*, the two species possessing very similar morphological characteristics both in vivo and after protargol impregnation [46]. *Oxytricha trifallax* was initially considered a synonym of *S. histriomuscorum* due to their similar morphology [46]. However, Zoller et al. [48] suggested that *O. trifallax* and *S. histriomuscorum* should be recognized as separate species since the two species have significant evolutionary differences. Küppers et al. [49] justified the transfer of *Sterkiella thompsoni* to a new genus *Parasterkiella* based on new data on the morphogenesis of the dorsal ciliature. Most recently, six new species, including *S. sinica* [14], *S. tetracirrata* [12], *S. subtropica* [11], *S. multicirrata* [13], *S. paratricirrata* [15], and *S. zhangi* [16], were reported, respectively. Therefore, with *S. thompsoni* removed to another genus *Parasterkiella*, the genus *Sterkiella* currently contains thirteen nominal species. Compared with the other species of the genus *Sterkiella*, only *S. subtropica*, *S. tricirrata*, *S. histriomuscorum*, *S. nova*, and *S. zhangi* have two macronuclei, so *S. histriomuscorum* can be separated from the other eight species by the number of macronuclei. *S. histriomuscorum* and *S. nova* are morphologically nearly indistinguishable, despite their genetic differences (Figure 7). However, *S. histriomuscorum* can be distinguished from *S. tricirrata* by the number of transverse cirri (4–5 in *S. histriomuscorum* vs. 3 in *S. tricirrata*) and dorsal kineties (6 vs. 5) [7], and is distinctly different from S. *subtropica* in its habitat (commonly in terrestrial and freshwater habitats vs. marine water, [11]), while it differs from *S. zhangi* in having a more average number of adoral membranelles (32 vs. 28) [7,16,46]. The morphological characteristics of our isolate are consistent with those of other *S. histriomuscorum* populations reported in the literature (Table S2), except the size of the macronucleus, which is much closer to the macronucleus size of the marine species *S. subtropica* [11]. We suggest that the difference was probably caused by the environmental habitat (natural lake or soil habitat for populations in the literature vs. BG11 medium for the present study).

For the molecular phylogenetic analysis, twenty SSU rRNA gene sequences from *Sterkiella* were analyzed, six of which were from *S. histriomuscorum*. In the phylogenetic tree (Figure 7), our isolate had the closest relationship with the *S. histriomuscorum* (HQ615720) identified by Zoller et al. [48]. However, considering that the different populations of *S. histriomuscorum* were separated and located in different clades, and the different species in the genus of *Sterkiella* were also separated and grouped with other genera in the subfamily of Stylonychinae, our results indicated that both the species of *S. histriomuscorum* and the genus of *Sterkiella* were not monophyletic, with these results being similar to those of molecular phylogenetic analyses on *Sterkiella* [11,12,13,14,15,16,18]. Since the inter-species and intra-species nucleotide differences were similar (Table 1), we speculate that SSU rRNA gene information alone is not enough, and the information from multiple genes or genomes is needed to shed light on the complicated phylogeny of the Stylonychinae.

### 4.2. Feeding Characteristics and Feeding Selectivity on the Microalgae

Numerous field studies have shown that microzooplankton are the predominant consumers of phytoplankton in aquatic ecosystems, among which planktonic ciliates are the most important algal consumers in many lakes and marine systems [50]. As herbivorous protistan grazers are diverse not only in terms of taxonomy but also in terms of size and feeding behavior [1], it is very important to clarify which group(s) of ciliates can graze algae and what their strategies are.

Recently, it was reported that microzooplanktonic grazers, including protistan taxa, pose a potentially devastating threat to the commercial success of microalgal mass culture [4]. For example, one species of chrysophyte, *Poterioochromonas malhamensis*, could cause the collapse of *Chlorella* culture [51], some vampyrellid amoebae could cause the collapse of *Scenedesmus* culture [27] and *Chlorella* culture [52], whereas heterolobosean amoeba *Euplaesiobystra perlucida* could cause the loss of *Phaeodactylum tricornutum* in pilot-scale cultures [53]. Especially, a comprehensive investigation into harmful microzooplankton species in mass cultures of a commercially promising species *Scenedesmus acuminatus* was conducted throughout the year and proved that the harmful grazers led to culture deterioration and reduced biomass yield [28]. In our study, we report for the first time that *S. histriomuscorum* has a strong ability to graze *Scenedesmus* cells in mass culture. In a previous study, only a small number of freshwater ciliates from eutrophic ponds, such as *Loxodes magnus*, *L. striatus*, *Frontonia leucas*, and *Stentor coeruleus*, have been reported as grazers of *Scenedesmus* [54]. However, the laboratory experiments showed that even though *L. magnus* can feed on *Scenedesmus* at a rate of 0.38 to 1.28 *Scenedesmus* cells/*L. magnus* per hour, it had no significant effect on the growth of *Scenedesmus*, and a very low rate of *Scenedesmus* cell division is sufficient to compensate for this loss. In contrast, our study showed that the grazing ability of *S. histriomuscorum* on *Scenedesmus* was substantially greater. As a member of Stylonychinea, *S. histriomuscorum* possesses a well-developed oral structure (Figure 2B) and grazes on *Scenedesmus* by active predatory hunting according to our microscopic observation, and the clearance rate of *Scenedesmus* could reach 87 cells per hour for one *S. histriomuscorum* cell. Perhaps there was an error in the clearance value because clumps of algae affect the counting value. It was found that the existence or grazing of *S. histriomuscorum* could induce the algal prey to actively form clumps, which is considered a defense strategy for algae [55]. The formation of algal clumps is a complicated process. Bacterial contamination in the culture or the feces of the ciliates enhances the formation of algal clumps, which is very common in outdoor algal cultures. The lab cultures are much cleaner, while the outdoor algal cultures are more likely to be contaminated and have more chances of forming clumps. Moreover, *Sterkiella* can graze *Scenedesmus* cells in a highly efficient way (Movie S1). Our TEM observations (Figure 5A) also revealed numerous large digestive vacuoles at different stages of the digestive process inside *S. histriomuscorum*, indicating its high digestive ability. The feeding characteristics of *S. histriomuscorum* are very similar to those of the ciliate *Pseudomicrothorax*, which has evolved a special oral structure and digestive mechanism to graze on filamentous cyanobacteria quickly and to digest the cell wall with special enzymes [56] and has been reported to play an important role in the control of algal blooms [57].

To graze on *Scenedesmus*, *S. histriomuscorum* must be able to break through the tough *Scenedesmus* cell wall, and we speculate that to do this, *S. histriomuscorum* must also possess a set of enzymes that can digest *Scenedesmus* cell walls efficiently. Many scientists are interested in making biofuels or extracting high-value products from algae; however, it is very difficult to break down the outer walls of algae to obtain energy-rich sugars. Perhaps the enzymes of algivorous predators may help resolve this problem. Future studies should focus on the extraction and application of such enzymes in algivorous predators. Moreover, since *Sterkiella* has an enormous ability to graze algae and *Sterkiella* can be cultured, we suggest that *Sterkiella* could serve as a model for studies of the relationship between an algivorous ciliate predator and unicellular algae.

In addition, it was reported that protozoa can effectively graze harmful algae and show strong tolerance to cyanobacteria toxins, e.g., *Paramecium* [58], *Ochromonas* [59], *Poterioochromonas malhamensis* [60] graze on *Microcystis*. However, our study revealed that *S. histriomuscorum* prefers chlorophyta as food and cannot graze on *Microcystis* or *Isochrysis.* Generally, size and shape remain first-order determinants of prey availability [1], but the nutritional quality of different microalgae seems to be more important for the grazer *Sterkiella.* The sizes of *Isochrysis* and *Nannochloropsis oceanica* are smaller than those of *Chlorella*, *Scenedesmus*, *Chlorogonium elongatum*, and *Chlamydomonas reinhardtii*, but they are not suitable food for *Sterkiella.* The high grazing capacity indicates that the nutritional qualities of *Chlorella* and *Scenedesmus* are more suitable for *Sterkiella* (Table 1). For the feeding selectivity experiment, only one genus of Cyanobacteria, *Microcystis*, was tested as prey. Therefore, other Cyanobacteria species and strains and other protozoan species could be tested in future studies to reveal more protozoan predators, which can potentially be used for the biological control of blooms caused by Cyanobacteria.

### 4.3. The Distribution of Sterkiella and the Function of the Cyst

*S. histriomuscorum* is one of the most widespread oxytrichids in natural environments [61], such as rivers [62] and soils [13], and recently, some species have been found in brackish [63] or marine water [11]. Compared with previous studies reported in the literature, our observations of *S. histriomuscorum* were from temporary water bodies: large-scale outdoor *Scenedesmus* cultures where *S. histriomuscorum* had a definite, negative effect on the growth of *Scenedesmus*.

The formation of resting cysts is a common survival strategy for many free-living ciliates [64,65]. As *S. histriomuscorum* never occurred in the indoor culture systems at the ASU facility and was found only in the outdoor culture systems, we speculate that *S. histriomuscorum* came from the environment, such as the air or the surrounding soil, where it existed as resting cysts. Similar results have been obtained for another herbivorous protozoan, *Poterioochromonas malhamensis*. We developed a qPCR method to detect the distribution of *P. malhamensis* in different environments and demonstrated that it likely entered the algal culture system from the air where it can exist as a cyst [66]. As Fenchel and Finlay [67] reported that most organisms smaller than 1 mm occur worldwide wherever their required habitats are realized, our study revealed that vegetative *S. histriomuscorum* cells could exist in *Scenedesmus* culture and graze *Scenedesmus* cells, which indicates that environmental transition from the air or soil to the culture medium could induce resting cysts to excyst. Under laboratory conditions, we found that vegetative cells readily formed cysts when the culture was depleted of algae (i.e., food); however, excystment of *S. histriomuscorum* could not be induced by the addition of new algal prey. By chance, when we isolated the cysts, we rinsed them with distilled water and then immersed them in distilled water at room temperature for about 10 min. We found that all the cysts became active and moved around, and this excystment phenomenon could easily be repeated. Perhaps immersion in distilled water can result in an osmotic pressure difference between the interior and exterior of the cysts, which stimulates the separation of the inner layer of the endocyst from the cytoplasm and ultimately induces the process of excystment.

Excystment is an important process by which a cyst becomes a vegetative cell. It seems that *S. histriomuscorum* has a range of different strategies for excystment. Adl and Berger [18] reported that cysts of *S. histriomuscorum* could be stimulated to excyst by transferring them from 17 °C distilled water to 27 °C with the addition of 4% (*v*/*v*) bacterized medium, and that rinsing with distilled water was essential to obtain a good yield, whereas Grisvard et al. [17] used 0.02% dried milk to induce excystment. Previous studies on the freshwater ciliate *Meseres corlissi* have also shown that the factors inducing en- and excystment differ between populations from geographically distant sites, as sometimes en- or excystment was controlled by ambient temperature [68], sometimes by water-soluble soil components, and sometimes could be induced by the addition of a soil extract to the culture medium [69].

On the basis of our observations and the studies reported in the literature, we can conclude that the encystment of *S. histriomuscorum* can be easily induced by food deprivation, but the food supply alone cannot stimulate excystment. The stimulus probably depends on the surrounding environment of the cysts: If the cyst exists in the air, water with soluble nutritional components could be the critical requirement; if the cyst exists in the culture medium, the composition and osmoticity of the medium and the food supply could both be stimulatory factors. Cysts have a strong multilayered wall that protects dormant cells from various environmental stresses, such as desiccation and predation [70]. However, during excystment, the ready-to-emerge ciliate must overcome the barrier of the multilayered cyst wall. In our study, cysts from the surrounding air or soil could excyst after entering the algal cultures, whereas newly formed cysts due to food shortage in the culture system were difficult to excyst unless they were transferred to an environment lacking ions, such as distilled water. The sharp change in the ion concentration between distilled water and the culture medium results in osmotic shock, which likely facilitates the excystment.

Therefore, we suggest that preventing the introduction of cysts into the culture system is the key to controlling the mass occurrence of *S. histriomuscorum* in the algal culture. On the one hand, this can be achieved by refreshing the air to reduce the density of cysts in the surrounding environment of the culture system; on the other hand, a fast detection method should be developed for *S. histriomuscorum* so that prompt control measures can be taken before outbreaks. In the future, additional research on excystment will be very helpful not only for improving our understanding of the ecological function of *S. histriomuscorum* in the natural food web but also for exploring ways to inhibit excystment, thereby reducing the grazing effect of *S. histriomuscorum* on the algal crop.

### 4.4. The Improvement of SEM Observation on S. histriomuscorum

In our study, examination of the morphological features by scanning electron microscopy was also not straightforward, proving to be very difficult to obtain good SEM results for *S. histriomuscorum*. The common problem encountered was that the cells were very fragile and easily broken. We tried several reagents to fix the samples, including 2% (working solution) glutaraldehyde or osmium acid, and finally found that if we added saturated mercuric chloride, the shape and organelles of the cells were retained very well. For our study, we found that the best results were achieved with a ratio of 1:1 osmium acid with a working solution of 2% to saturated mercuric chloride, compared to the ratio of 1:6 reported by Gu and Ni [71]. This fixing method should also be applicable to other species of the genus *Sterkiella*.

## 5. Conclusions

The results of our study provide new data to unravel the confusing taxonomy and feeding characteristics of the well-known herbivorous hypotrich ciliate *Sterkiella histriomuscorum.* A full description was provided by combining morphological and molecular phylogenetic analyses, as well as an analysis of feeding. Our study reported a special method to induce the cysts of *S. histriomuscorum* to excyst and revealed that *S. histriomuscorum* prefers to graze on chlorophytes and the diatom *Phaeodactylum tricornutum* but not on chrysophytes or cyanobacteria. Moreover, phylogenetic analysis indicated that both the genus *Sterkiella* and the species *S. histriomuscorum* are non-monophyletic. This study improves the understanding of the biology, morphology, systematics, and ecological function of *S. histriomuscorum*, and could also be very useful in the development of an early warning system and control measures for preventing or treating this contaminant in microalgal mass cultures.

## Figures and Tables

**Table 1:** Potential capacity of *Sterkiella histriomuscorum* to prey upon various commercial microalgae. The microalgal strains were classified into “most suitable/rapid growth (++)”, “suitable limited growth (+)”, and “not suitable/no growth (-)” categories according to the ability of *S. histriomuscorum* to feed on prey and grow.

Food Group	Algal Species	Strain	Morphology	Cell Size (µm)	Algal Category
Chlorophyte	*Chlorella vulgaris*	FACHB-8	unicellular	2–3	++
	*Chlorella pyrenoidosa*	FACHB-9	unicellular	2–3	++
	*Chlorella sorokiniana*	CGMCC11801	unicellular	3–4	++
	*Scenedesmus acuminatus*	CMBB154	unicellular	2–3 × 8–10	++
	*Chlorogonium elongatum*	CMBB132	unicellular	2–3 × 6–8	++
	*Chlamydomonas reinhardtii*	CC124	unicellular	7–10	++
	*Chlorella sorokiniana*	FACHB-275	unicellular	5–6	+
	*Chlorella sorokiniana*	CMBB146	unicellular	3–8	+
	*Nannochloropsis oceanica*	IMET1	unicellular	2–4	-
	*Ulothrix* sp.	FACHB-1747	filamentous	1–2 × 10–15	-
Chrysophyte	*Isochrysis zhanjiangensis*	CMBB282	unicellular	5–6 × 6–7	-
	*Isochrysis* sp.	CMBB112	unicellular	3–4 × 4–5	-
Diatom	*Phaeodactylum tricornutum*	UTEX640	unicellular	3–4 × 10–15	++
Cyanobacteria	*Microcystis aeruginosa*	FACHB-928	unicellular	2–3	-
	*Microcystis aeruginosa*	FACHB-905	unicellular	2–3	-
	*Microcystis flos-aquae*	FACHB-1028	unicellular	2–3	-
	*Microcystis aeruginosa*	FACHB-942	unicellular	2–3	-

**Table 2:** Morphometric data on *Sterkiella histriomuscorum* (unit: μm) based on protargol-impregnated specimens.

Characteristics	Min	Max	Mean	SD	CV (%)	n
Body length	93	138	110	12.16	0.11	30
Body width	35	60	47	8.39	0.18	30
Adoral zone, length	34	52	42	5.20	0.13	30
Adoral membranelles, No.	28	38	32	2.76	0.09	30
Buccal cirri, No.	1	1	1	0	0	30
Frontal cirri, No.	3	3	3	0	0	30
Frontoventral cirri, No.	3	4	4	0.18	0.05	30
Posterior ventral cirri, No.	3	3	3	0	0	16
Pretransverse ventral cirri, No.	2	2	2	0	0	16
Transverse cirri, No.	4	5	5	0.41	0.08	30
Cirri in left marginal row, No.	18	25	21	2.16	0.10	30
Cirri in right marginal row, No.	22	27	25	1.27	0.05	30
Caudal cirri, No.	3	3	3	0	0	16
Dorsal kineties, No.	5	7	6	0.62	0.11	16
Macronuclear nodules, No.	2	2	2	0	0	30
Macronuclear nodule, length	25	42	32	4.24	0.13	50
Macronuclear nodule, width	18	30	23	3.17	0.14	50
Micronuclear nodules, No.	1	3	2	0.78	0.41	30

## Data Availability

The original contributions presented in this study are included in the article/Supplementary Materials. Further inquiries can be directed to the corresponding authors.

## Supplementary Materials

The following supporting information can be downloaded at: https://www.mdpi.com/article/10.3390/microorganisms13051016/s1. Table S1: Numbers of unmatched nucleotides (upper right) and sequence identities (lower left) between the SSU rDNA of five species of the genus Sterkiella (ten populations). * indicates the strain sequenced in our study; Table S2: Morphometric characteristics of populations of *Sterkiella histriomuscorum* reported in this and other studies; Movie S1: Feeding process of *S. histriomuscorum* on *Scenedesmus* cells. References [9,10,61,72,73] are cited in the supplementary materials.
