# Supplementary material for: Identification and Feeding Characterization of Sterkiella histriomuscorum (Protozoa, Ciliophora, Hypotrichia) Isolated from Outdoor Mass Culture of Scenedesmus dimorphus
Source: Microorganisms. 2025 Apr 28;13(5):1016. doi: 10.3390/microorganisms13051016 (PMC12114034; doi:10.3390/microorganisms13051016)
Supplement: Supplementary file 1 [file microorganisms-13-01016-s001.zip › supplementary materials.pdf]

**Supplementary Materials:**

**Table S1.** Numbers of unmatched nucleotides (upper right) and sequence identities (lower left) between the SSU rDNA of five species of the genus *Sterkiella* (ten populations). * indicates the strain sequenced in our study.

| | Sp1 | Sp2* | Sp3 | Sp4 | Sp5 | Sp6 | Sp7 | Sp8 | Sp9 | Sp10 |
|---|---|---|---|---|---|---|---|---|---|---|
| Sp1 *S.histriomuscorum* (HQ615720) | 0 | 1 | 19 | 19 | 18 | 17 | 12 | 22 | 13 | 21 |
| Sp2 * *S. histriomuscorum* (KX355209) | 0.999 | 0 | 20 | 20 | 19 | 18 | 13 | 23 | 14 | 22 |
| Sp3 *S. histriomuscorum* (AF508770) | 0.987 | 0.985 | 0 | 0 | 1 | 1 | 7 | 21 | 17 | 30 |
| Sp4 *S. histriomuscorum* (FJ545743) | 0.987 | 0.985 | 1.000 | 0 | 1 | 1 | 7 | 21 | 17 | 30 |
| Sp5 *S. histriomuscorum* (KC193240) | 0.988 | 0.986 | 0.999 | 0.999 | 0 | 2 | 6 | 22 | 18 | 31 |
| Sp6 *S. histriomuscorum* (GU942566) | 0.988 | 0.986 | 0.998 | 0.998 | 0.998 | 0 | 6 | 22 | 16 | 36 |
| Sp7 *S. cavicola* (GU942565) | 0.991 | 0.989 | 0.996 | 0.996 | 0.996 | 0.995 | 0 | 22 | 16 | 31 |
| Sp8 *S. subtropica* (KM924307) | 0.985 | 0.983 | 0.987 | 0.987 | 0.986 | 0.985 | 0.986 | 0 | 21 | 31 |
| Sp9 *S. tetracirrata* (KF668619) | 0.991 | 0.989 | 0.989 | 0.989 | 0.989 | 0.989 | 0.99 | 0.987 | 0 | 22 |
| Sp10 *S. nova* (AF508771) | 0.986 | 0.984 | 0.981 | 0.981 | 0.981 | 0.976 | 0.981 | 0.981 | 0.986 | 0 |

**Table S2.** Morphometric characteristics of populations of *Sterkiella histriomuscorum* reported in this and other studies.

| Source | BL (μm) | BW (μm) | AZM, length (μm) | AZM no. | FC no. | VC no. | TC no. | LMR no. | RMR no. | CC no. | DK no. | MA Length (μm) | MA Width (μm) |
|---|---|---|---|---|---|---|---|---|---|---|---|---|---|
| [1] | 57-75 | 28-42 | 25-32 | 27-32 | | | 4-5 | 12-18 | 17-21 | | | | |
| [2] | 85-129 | 41-62 | 39-53 | 34-44 | | | 4-6 | 18-25 | 24-32 | | | | |
| [3] | 100-114 | 47-59 | 41-47 | 27-31 | | | 4 | 15-20 | 20-23 | | | | |
| [4] | 66-102 | 35-57 | 31-45 | 26-31 | 8 | 5 | 5 | 15-21 | 18-22 | 3 | 5-6 | 14-29 | 8-15 |
| [5] | 85-160 | 42-85 | 35-52 | 29-38 | 8 | 5 | 3-5 | 17-23 | 20-24 | 3 | 6 | 15-20 | 10-15 |
| Current study | 93-138 | 35-60 | 34-52 | 28-38 | 7-8 | 5 | 4-5 | 18-25 | 22-27 | 3 | 5-7 | 25-42 | 18-22 |

BL: Body length; BW: Body width; AZM: Adoral zone, length; FC: Frontal cirri; VC: Ventral cirri; TC: Transverse cirri; LMR: Cirri in left marginal row; RMR: Cirri in right marginal row; CC: Caudal cirri; DK: Dorsal kineties; MA: Macronuclear nodule.

## References

1. 11 Berger, H.; Foissner, W.; Adam, H. Morphological variation and comparative analysis of morphogenesis in *Parakahliella macrostoma* (Foissner, 1982) nov. gen. and *Histriculus muscorum* (Kahl, 1932), (Ciliophora, Hypotrichida). *Protistologica (Paris)* **1985**, *21*, 295–311.
2. 10 Augustin, H.; Foissner, W. Morphologie und Ökologie einiger Ciliaten (Protozoa: Ciliophora) aus dem Belebtschlamm. *Archiv fuer Protistenkunde* **1992**, *141*, 243–283.
3. 14 Shin, M.K.; Kim, W. Morphology and biometry of two oxytrichid species of genus *Histriculus* Corliss, 1960 (Ciliophora, Hypotrichida, Oxytrichidae) from Seoul, Korea. *Korean Journal of Zoology* **1994**, *37*,113–119.
4. 13 Petz, W.; Foissner, W. Morphology and infraciliature of some soil ciliates (Protozoa, Ciliophora) from continental Antarctica, with notes on the morphogenesis of *Sterkiella histriomuscorum*. *Polar Record* **1997**, *33*, 307–326.
5. 12 Jiang, J.M.; Ma, H.G.; Shao, C. Morphology and morphogenesis of *Sterkiella histriomuscorum* (Ciliophora, Hypotricha). *Acta Hydrobiologica Sinica* **2013**, *37*, 227–234.
